# Perspectives and Experiences of Doctors and Pharmacists on the Clinical Use of Direct Oral Anticoagulants in Saudi Arabia

**DOI:** 10.3390/pharmacy14010021

**Published:** 2026-02-02

**Authors:** Dalal Salem Aldossari, Komal Latif, Amjad Nasser Alsadoni, Orjuwan Hasan Alshehri, Rakan Ibrahim Binjathlan, Monirah Mutlaq Alenezy, Taif Farhan Alshahrani, Hana Ahmed Lubbad, Rana Saeed Alshamasi, Abdulmajead Khaled Alanazi, Raed Ghazi Alotaibi, Ghazi Ibrahim Arishi, Sheraz Ali

**Affiliations:** 1 Pharmaceutical Care Services, King Saud Medical City, Ministry of Health, Riyadh 12372, Saudi Arabia; 2 Institute of Public Health and Clinical Nutrition, Faculty of Health Sciences, University of Eastern Finland, 70210 Kuopio, Finland; 3 Samta General Hospital, Jazan Health Cluster, Ministry of Health, Samtah 86735, Saudi Arabia; 4 Institute for Evidence-Based Healthcare, Bond University, Robina, QLD 4226, Australia; 5 School of Dentistry and Medical Sciences, Faculty of Science and Health, Charles Sturt University, Orange, NSW 2800, Australia

**Keywords:** direct oral anticoagulants, DOACs, physicians, pharmacists, clinical practice, anticoagulation therapy, healthcare professionals, perspectives, experiences

## Abstract

Background and objectives: Research into clinicians’ and pharmacists’ experiences and perspectives on direct oral anticoagulant (DOAC) use in Saudi Arabia and the broader Middle Eastern area is limited. Therefore, we aimed to evaluate the perspectives and experiences of physicians and pharmacists practicing in Saudi Arabia who prescribe DOACs and dispense DOAC therapy, respectively. Methods: A cross-sectional study was undertaken utilizing an online survey instrument. We collected data via Google Forms. Between June and July 2024, the study questionnaire was distributed to community pharmacists, general practitioners [GPs], cardiologists, residents in internal medicine, and hospital pharmacists (primary and secondary healthcare professionals) working in Saudi Arabia. Results: Comprising 146 doctors and 167 pharmacists, 313 total healthcare professionals participated in the study. Of the weekly DOAC prescriptions, cardiologists had the most at 35%; internal medicine residents came next at 16.3% and general practitioners at 17.5%. Among pharmacists, 16.7% of community pharmacists and 23.9% of hospital pharmacists dispensed DOACs weekly. The most often prescribed and dispensed medications were rivaroxaban, edoxaban, and apixaban. Across all categories, Lexicomp was the most often used tool. Most physicians (98%) said they lowered the DOAC dose when necessary. Especially in dosing, preoperative care, patient education, and medication interaction identification, internal medicine residents and hospital pharmacists expressed more confidence in managing DOACs. In these domains, community pharmacists expressed less trust. Conclusions: This study revealed that most participants preferred newer oral anticoagulants over warfarin and demonstrated a fairly good level of self-perceived knowledge regarding various aspects of the clinical use of DOACs. The study findings highlight the importance of focused training initiatives to standardize the use of DOACs, boost trust among community pharmacists and GPs, and ensure safe and effective patient care.

## 1. Introduction

The last decade has witnessed the introduction of four direct oral anticoagulants (DOACs): dabigatran, rivaroxaban, apixaban, and edoxaban. DOACs are prescribed to patients with non-valvular atrial fibrillation (AF) for the prevention and treatment of venous thromboembolism (VTE) and to mitigate the risk of stroke and systemic embolism [1,2,3,4,5]. DOACs were developed to overcome the limitations of vitamin K antagonists (VKAs), which had been the only effective oral anticoagulants for over five decades. DOACs, as opposed to VKAs, are intended for fixed-dose administration without requiring routine coagulation monitoring; they also exhibit a quicker onset of action and a reduced propensity for food–drug and drug–drug interactions (DDIs). Additionally, they have demonstrated no inferiority or even superiority to VKAs and are linked to reduced intracranial haemorrhage [1,2,3,5]. International guidelines have therefore indicated a preference for DOACs as opposed to VKAs [6,7,8]. As a result, anticoagulation therapy underwent a paradigm shift, and DOACs surpassed VKAs in usage [9].

However, research shows that DOACs are frequently prescribed inappropriately, which increases the risk of medicine-related harm [10,11]. Furthermore, individuals diagnosed with AF are usually older and present with comorbidities and polypharmacy [6]. As a result, a diverse range of healthcare professionals provide care and follow-up for many of these patients. Stroke prevention in atrial fibrillation patients is of paramount importance [12]; hence, anticoagulation must be prescribed appropriately, and the patient must adhere to their prescribed medication regimen [13]. Assessing and enhancing the proper use of anticoagulants is crucial for optimal care of patients with atrial fibrillation, and this responsibility falls on several healthcare providers, including physicians and pharmacists [14,15]. The integrated management of patients undergoing anticoagulant therapy, which includes collaborative decision making and a structured multidisciplinary approach, is recommended by international guidelines [6]. This integrated care approach in AF has been shown to be associated with decreased cardiovascular hospitalizations and all-cause mortality [16].

A previous review by Generalova et al. revealed that, despite the increasing usage of DOACs globally, there is a lack of evidence on clinicians’ perspectives and experiences on DOAC use [17]. However, such evidence is crucial, for instance, for the development and implementation of future healthcare strategies that assist various healthcare professionals in fulfilling their responsibility to optimize DOAC utilization. Research into clinicians’ and pharmacists’ experiences and perspectives on DOAC use in Saudi Arabia and the broader Middle Eastern area is limited. Therefore, this study aimed at exploring how clinicians and pharmacists use direct oral anticoagulants in routine practice, with particular focus on prescribing behavior from the physicians’ side, self-perceived knowledge, and views compared with vitamin K antagonists among physicians and pharmacists.

## 2. Methods

### 2.1. Study Design and Setting

A cross-sectional study was undertaken utilizing an online survey instrument. We collected data via Google Forms. Between June and July 2024, the study questionnaire was distributed to primary healthcare professionals (community pharmacists, general practitioners [GPs]) and secondary healthcare professionals (cardiologists, residents in internal medicine, and hospital pharmacists) working in Saudi Arabia. As of 2024, Saudi Arabia had more than 500 hospitals with more than 40 specialized centers providing services to patients with heart disease, while many secondary and tertiary hospitals provide general cardiology care.

### 2.2. Study Tool

The survey was adopted from a previous study [18] with some changes to make it simpler and more practical for our study. Unlike the original, which included detailed clinical cases of patients with atrial fibrillation, we removed the cases to make the survey quicker and easier to complete. We also reworded some questions for clarity. To make sure these changes did not affect the survey’s validity, we piloted it with ten healthcare professionals. Their feedback helped us improve clarity, ensure the questions were easy to answer, and confirmed that the survey could be completed in about ten minutes.

The questionnaire was structured into four distinct sections. The first section, titled “Current Practice,” delved into the respondents’ existing practices regarding the use of DOACs. The second section, “Prescribing Behaviour,” explored the respondents’ habits and behaviors when prescribing DOACs. The third section, “Self-Perceived Knowledge About DOACs,” aimed to gauge how respondents view their own understanding and knowledge of DOACs. The last section, “Views and Opinions About DOACs Vs. VKAs,” captured the respondents’ perspectives and opinions comparing DOACs with VKAs. Some survey items were framed differently for physicians and pharmacists to reflect their distinct roles. Physicians were asked to rate the statement, ‘I have sufficient knowledge to prescribe DOACs at the appropriate dose’ (doctors only), while pharmacists were asked to rate, ‘I have sufficient knowledge to dispense DOACs at the appropriate dose’ (pharmacists only). The survey cover page included a brief information sheet for prospective study participants.

### 2.3. Recruitment and Sample Size

An email invitation to participate in the online survey was distributed to the cardiology departments of hospitals in Saudi Arabia. Through their department heads, cardiologists were invited to participate in the online survey. The study team contacted the GPs and pharmacists in the following manner: initially, the researchers disseminated the survey link to individuals in their personal network, including friends, family members, coworkers, and neighbours, through popular social media platforms such as Facebook, LinkedIn, X, and WhatsApp. These participants were also requested to distribute this survey to colleagues through social media platforms. A reminder email was sent after one week. Because invitations were distributed through intermediaries and multiple open channels, the total number of individuals who received or viewed the survey invitation could not be determined. As a result, a formal response rate could not be calculated.

All physicians (regardless of medical specialty) are authorized to prescribe DOACs in Saudi Arabia. DOACs are dispensed to hospitalized and ambulatory patients, respectively, by hospital pharmacists and community pharmacists; they are not authorized to prescribe DOACs. The Saudi Arabian Ministry of Health Portal data for the year 2020 indicated that the aggregate count of medical doctors and pharmacists in Saudi Arabia amounted to 122,865 [19]. A target sample size of 383 was estimated using the Raosoft calculator, considering the population size, a 95% confidence level, and a 5% margin of error. Due to practical constraints and the voluntary nature of participation, 313 healthcare professionals were ultimately recruited, representing about 82% of the intended sample.

### 2.4. Ethical Considerations

This study was approved by the King Saud Medical City Institutional Review Board (IRB) with reference number H-01-R-053 on 1 July 2024. The completion of the survey was considered as implied consent.

### 2.5. Inclusion and Exclusion Criteria

#### 2.5.1. Inclusion Criteria

Licensed physicians or pharmacists currently in practice in Saudi Arabia with at least one year of practice and who were involved in the prescribing and dispensing of oral anticoagulants.

#### 2.5.2. Exclusion Criteria

Students, incomplete responses, and duplicate entries (same IP address/devices).

### 2.6. Data Analysis

All statistical analyses were conducted using IBM SPSS Statistics version 27. For continuous variables, the data were presented as mean ± standard deviation if they followed a normal distribution or as median (interquartile range) if they did not. Additionally, graphical representation was used to represent the different scenarios. For analysis, cardiologists and internal medicine residents were grouped as “specialists,” while general practitioners were kept as a separate category. This was based on care setting and clinical role rather than seniority, as both cardiologists and residents work in hospital settings and are involved in starting and managing DOAC therapy in inpatients. In routine practice, residents usually prescribe under the supervision of senior physicians, so their decisions largely follow specialist judgment and hospital protocols. Similarly, for analysis, prescribing frequency was collapsed into two categories: “frequent” (monthly, weekly, or daily) and “infrequent” (never or annually). This was done to distinguish clinicians with regular exposure to DOAC prescribing from those with minimal exposure. We acknowledge that monthly and daily prescribing do not represent the same level of experience and that this categorization reduces clinical nuance; original frequency categories are therefore presented in descriptive tables as supplementary tables.

We started with 313 survey participants. For each analysis, we only included responses that were complete and consistent. Any incomplete or invalid responses were left out, which is why the number of participants shown in some tables is slightly lower than the total.

## 3. Results

A total of 313 healthcare professionals participated in the survey, including 146 physicians (40 cardiologists, 49 internal medicine residents, and 57 general practitioners) and 167 pharmacists (113 hospital pharmacists and 54 community pharmacists). More than three-fourths (76.4%) of participants had clinical experience of less than 15 years.

### 3.1. Practice and Experiences with DOACs

Both pharmacists (both community and hospital) and physicians (specialists and GPs) were asked how frequently they dispense or prescribe DOACs. Accordingly, DOACs prescribing/dispensing was predominantly frequent across all groups. In hospital pharmacies, 84.1% of DOACs were dispensed frequently, compared to 91.1% in community pharmacies, with no significant difference (χ^2^ = 1.33, *p* = 0.249; Fisher’s exact *p* = 0.315). Similarly, both specialists (cardiologists and internal medicine residents) and general practitioners reported frequent DOAC prescribing in over 93% of cases with no statistically significant differences (χ^2^ = 0.004, *p* = 0.949; Fisher’s exact *p* = 0.1.000). Overall, frequent DOAC dispensing and prescribing is the dominant pattern, independent of pharmacy setting or physician type (see Table 1).

Participants were also asked to indicate all the DOACs they commonly prescribe or dispense. Accordingly, among 304 valid cases, apixaban was the most commonly prescribed/dispensed DOAC in both primary (83%) and secondary care (84%), with no significant difference between settings (χ^2^ = 0.005, *p* = 0.94). Edoxaban prescription/dispensing was higher in secondary care (58%) than primary care (42%), and this difference was statistically significant (χ^2^ = 5.10, *p* = 0.024). Rivaroxaban and dabigatran were less frequently used, and their distribution did not differ significantly between care levels. Overall, apixaban dominated prescribing and dispensing, edoxaban was more common in secondary care, and the remaining DOACs showed minimal variation by setting (see Table 2).

Reduced-dose DOAC prescription or dispensing was most commonly reported as intermittent among both professional groups. Among physicians, 41.8% reported sometimes prescribing and 32.8% often prescribing reduced-dose DOACs, whereas 36.4% of pharmacists reported sometimes dispensing and 33.8% often dispensing them. Infrequent use (never or rarely) was reported by 12.7% of physicians and 19.2% of pharmacists, while frequent prescription or dispensing of reduced doses of DOACs (often or always) was comparable between physicians (45.5%) and pharmacists (44.4%) (see Table 3).

Prescribers were also asked about some of the factors that may influence their prescription behavior. Accordingly, among the 146 doctors surveyed, clinical factors were the main drivers of DOAC-prescribing decisions. Most respondents considered renal function and the drug’s side effect profile to be important, while patient age and body weight were also influential for a substantial number. In comparison, personal experience with a specific DOAC, patient preference, and information from medical representatives were less frequently cited as important. Overall, this pattern suggests that specialists rely primarily on objective patient-related clinical factors when selecting a DOAC, rather than experiential or external influences (see Table 4).

### 3.2. Use of Guidelines and Resources

**Figure 1:**
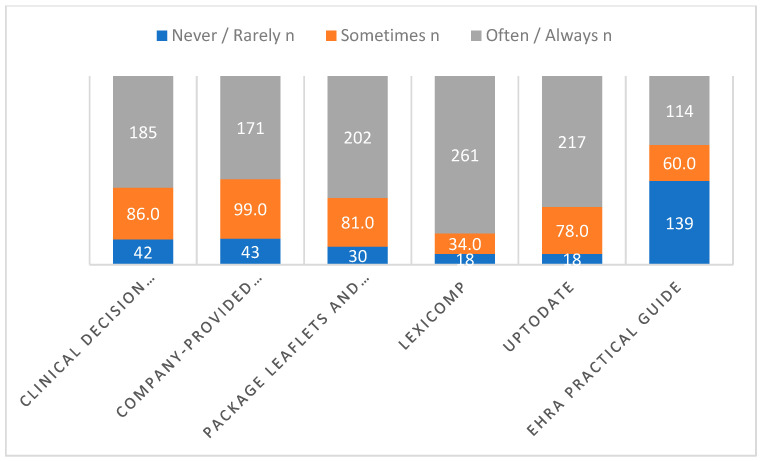
Frequency of use of different guidelines and resources by study participants when prescribing or dispensing.

The following figure shows how often study participants use different sources to guide DOAC prescribing and dispensing. Digital tools like Lexicomp and UpToDate, along with package leaflets, are used most frequently, with the majority consulting them “often” or “always.” Clinical decision support systems and company brochures are also used regularly, though slightly less. In contrast, the EHRA Practical Guide is referred to less often, with nearly half of respondents reporting “never” or “rarely” using it. Overall, this suggests that clinicians rely more on readily accessible digital and regulatory sources than on formal guideline documents (see Figure 1).

### 3.3. Self-Perceived Knowledge

Participants were also asked to rate their level of agreement about their perceived knowledge of DOACs across four domains. Most respondents felt confident in perioperative management (83% of physicians, 75% of pharmacists) and drug–drug interactions (80% vs. 84%). Self-perceived knowledge of patient counseling and contraindications was slightly lower, with 82–83% agreement and higher uncertainty among pharmacists. Overall, both groups feel competent in clinical management, though gaps remain in patient-facing counseling and contraindication self-reported knowledge (Table 5).

On the other hand, participants were also asked whether they have sufficient knowledge to prescribe (physicians only) and dispense (pharmacists only) DOACs at the appropriate dose. Overall, self-reported confidence was high across all groups. Nearly all general practitioners reported agreement or strong agreement (95.9%), compared with 81.8% of specialists, among whom uncertainty and disagreement each accounted for 9.1%. Among pharmacists, agreement remained common in both settings (83.3% in hospital vs. 80.5% in community), although uncertainty was more frequent in community pharmacists (16.7%) than hospital pharmacists (11.5%) (Table 6).

### 3.4. Perception Towards DOACs and Vitamin K Antagonists

Participants were also asked to reflect on their opinion towards DOACs vs. Vitamin K antagonists. Both physicians and pharmacists generally favored DOACs over VKAs. For example, 91% of physicians and 68% of pharmacists agreed that DOACs have fewer side effects, while 86% of physicians and 75% of pharmacists preferred DOACs overall. Pharmacists were more confident across most items, including DOACs’ effectiveness (82% vs. 68%), adherence (82% vs. 65%), and the advantage of no INR monitoring (73% vs. 41%). Physicians showed more uncertainty, particularly on bleeding risks and in frail or renal failure patients, where 42% favored VKAs in renal failure compared to 24% of pharmacists. Several of these differences were statistically significant (Table 7).

## 4. Discussion

DOACs are a great step forward in the field of anticoagulation. They are getting preference over VKAs in the guidelines for various indications requiring anticoagulation [20]. The current study aimed to explore prescribers’ and pharmacists’ perspectives and experiences in managing newer direct-acting oral anticoagulants in clinical practice in Saudi Arabia, along with their self-assessed knowledge on various aspects of these medicines.

In the present study, prescribing frequency was compared between clinicians working in secondary care (cardiologists and internal medicine residents) and those in primary care (general practitioners), with no significant difference observed (93.3% vs. 93.0%; χ^2^ test, *p* > 0.05). In contrast, the Belgian study by Capiau et al. compared cardiologists directly with internal medicine residents and general practitioners, reporting more frequent DOAC prescribing among cardiologists [18]. This apparent discrepancy is likely methodological rather than substantive, reflecting differences in comparator groups and healthcare settings, as cardiologists are more often involved in DOAC initiation and complex anticoagulation decisions, while maintenance prescribing is now routinely shared across levels of care.

Prescribers and pharmacists were asked to report how often reduced-dose DOACs were prescribed or dispensed using a five-point Likert scale. Most physicians reported prescribing reduced doses at least sometimes, with 41.8% indicating “sometimes,” 32.8% “often,” and 12.7% “always,” while only a small proportion reported rarely or never doing so. A similar pattern was observed among pharmacists, where reduced-dose DOAC dispensing was reported as “sometimes” or more frequent by the majority of respondents. Together, these findings indicate that DOAC dose reduction is a routine and well-integrated practice in clinical care. This is consistent with reports from Belgium, where cardiologists, internal medicine residents, and general practitioners estimated that approximately one-quarter to one-third of their patients received reduced-dose DOACs [18]. Clinically, this likely reflects the high prevalence of multimorbidity among patients receiving anticoagulation, many of whom meet guideline-recommended dose reduction criteria related to age, renal function, body weight, or bleeding risk [21].

In this study, clinicians drew on a mix of information sources when making decisions about DOAC prescribing and dispensing. In day-to-day practice, quick, point-of-care tools were clearly favored. Resources such as Lexicomp, clinical decision support systems, and package leaflets were most often used, while UpToDate was consulted regularly but not consistently by all participants. By contrast, the EHRA Practical Guide was used less frequently, with many respondents reporting only occasional or rare consultation. This contrasts with findings from Belgium, where cardiologists relied more heavily on the EHRA guidance [18]. The difference likely reflects practical realities rather than disagreement with the guidance itself—clinicians tend to favor resources they know well, can access easily, and can apply rapidly in busy clinical settings, especially when managing complex patients on anticoagulation.

Patient preference was the most frequently cited factor influencing the prescription of a specific DOAC across all three specialties of prescribers, followed by the side effect profile, renal function, weight, and individual drug characteristics.

Most physicians and pharmacists in this study reported self-reported confidence in their knowledge of DOAC use, including perioperative management, patient counselling, management of drug–drug interactions, and recognition of contraindications. Around four in five respondents agreed that they were sufficiently knowledgeable across these domains. Self-reported confidence in appropriate dosing was also high, particularly among general practitioners, although a small proportion of specialists and hospital pharmacists reported uncertainty. These findings are in line with reports from Saudi Arabia showing generally good knowledge of DOAC use among prescribers [22]. Nevertheless, the presence of uncertainty among a subset of participants highlights the need for ongoing, targeted training to support safe and consistent DOAC use across clinical settings.

The study also highlighted that prescribers and pharmacists generally had a positive self-perception of their knowledge in key areas, including appropriate dosing, patient education, identifying and managing drug–drug interactions, and contraindications. The majority of participants—over two-thirds—believed they possessed sufficient knowledge in these aspects. A lack of knowledge in these areas can lead to medication errors. A previous cross-sectional cohort study conducted in Saudi Arabia highlighted several issues with newer anticoagulants. These included incorrect initial doses, inappropriate maintenance doses, and potentially significant drug–drug interactions, among other concerns [23]. A knowledge gap regarding the use of oral anticoagulants (OACs) was identified as one of the contributing factors in a qualitative study involving prescribers in Saudi Arabia [24]. Consistent with this, a systematic review and meta-analysis on contributory factors related to medication errors in this group of medicines identified knowledge gaps as one of the primary factors [25]. This finding underscores the importance of assessing the actual knowledge levels of practitioners and implementing appropriate educational interventions accordingly.

Lastly, we looked at some of our findings in light of the results reported by Capiau et al. in Belgium to give an international perspective. These comparisons are meant as a reference rather than a direct benchmark, because healthcare systems, prescribing habits, and patient populations in Saudi Arabia and Belgium are quite different. Such differences can affect how DOACs are prescribed, dose-adjusted, and monitored in everyday practice. So, while the Belgian study gives useful context, our results are best understood within the local healthcare setting.

### Study Limitations

As we utilized an online survey as our methodology, it may be subject to social desirability bias, where respondents tend to provide more favourable answers to knowledge-based questions. Consequently, the reported results may not accurately reflect their actual level of knowledge. Although technical measures were used to limit multiple submissions from the same device and responses were screened for obvious duplicates during data cleaning, the possibility of the same individual completing the survey more than once cannot be fully excluded. This may have led to a modest inflation of the sample size or minor distortion in the distribution of responses across professional groups. Furthermore, other professionals including surgeons and orthopedics were not involved, limiting the generalizability of the result. In addition, the use of different recruitment strategies across professional groups may have introduced selection bias.

## 5. Conclusions

This study revealed that most participants preferred newer oral anticoagulants over warfarin, and participants expressed confidence in their knowledge regarding various aspects of the clinical use of DOACs. The study findings highlight the importance of focused training initiatives to standardize the use of DOACs, boost trust among community pharmacists and GPs, and ensure safe and effective patient care. Qualitative research is recommended to ascertain the factors affecting healthcare professional (HCP) adherence to DOAC guidelines, particularly those with lower self-reported confidence levels.

## Figures and Tables

**Table 1:** DOAC dispensing and prescribing frequencies across pharmacies (N = 158) and physician (N = 146) groups.

	Group	Infrequent	Frequent	Total (n)	
Pharmacy setting	Hospital	18 (15.9%)	95 (84.1%)	113	χ^2^ (df = 1) = 1.33, *p*-value = 0.249, Fisher’s exact *p* = 0.315
	Community	4 (8.9%)	41 (91.1%)	45	
	Total	22 (13.9%)	136 (86.1%)	158	
Physicians	Specialists (cardiologists + internal medicine residents)	6 (6.7%)	83 (93.3%)	89	χ^2^ (df = 1) = 0.004, *p*-value = 0.949, Fisher’s exact *p* = 1
	General practitioners	4 (7.0%)	53 (93.0%)	57	
	Total	10 (6.8%)	136 (93.2%)	146	

**Table 2:** DOAC prescription/dispensing among professionals across primary and secondary care settings (N = 304).

DOAC	Primary Care *–Yes n (%)	Primary Care–No n (%)	Secondary Care **–Yes n (%)	Secondary Care–No n (%)	Total n	χ^2^ (df = 1)	*p*-Value (Chi-Square)	Fisher’s Exact *p*
Apixaban	85 (83.3%)	17 (16.7%)	169 (83.7%)	33 (16.3%)	304	0.005	0.942	1.000
Edoxaban	43 (42.2%)	59 (57.8%)	59 (57.8%)	143 (70.8%)	304	5.097	**0.024**	0.029
Rivaroxaban	33 (32.4%)	69 (67.6%)	71 (68.3%)	131 (65.5%)	304	0.235	0.628	0.701
Dabigatran	17 (16.7%)	85 (83.3%)	28 (13.9%)	174 (86.1%)	304	0.423	0.515	0.608

* Professionals in primary care include general practitioners and community pharmacists. ** Professionals in secondary care include cardiologists and internal medicine residents.

**Table 3:** Frequency of reduced-dose DOAC prescribing/dispensing by professional group.

Frequency of Reduced-Dose DOAC Prescription/Dispensing	Physicians (n = 134)	Pharmacists (n = 151)
Never	6 (4.5%)	5 (3.3%)
Rarely	11 (8.2%)	24 (15.9%)
Sometimes	56 (41.8%)	55 (36.4%)
Often	44 (32.8%)	51 (33.8%)
Always	17 (12.7%)	16 (10.6%)

**Table 4:** Physicians’ perspectives on factors influencing DOAC-prescribing decisions.

Response Category	The Patient’s Age N (%)	The Patient’s Body Weight N (%)	The Patient’s Renal Function N (%)	My Personal Experience with a Specific DOAC N (%)	The Side Effect Profile of a DOAC N (%)	Information Provided by the Company’s Medical Representative Influences N (%)	The Patient’s Personal Preference N (%)
Strongly disagree	14 (9.6%)	15 (10.3%)	8 (5.5%)	24 (16.4%)	4 (2.7%)	5 (3.4%)	20 (13.7%)
Disagree	21 (14.4%)	19 (13.0%)	2 (1.4%)	16 (11.0%)	2 (1.4%)	15 (10.3%)	17 (11.6%)
Neutral	9 (6.2%)	10 (6.8%)	15 (10.3%)	15 (10.3%)	11 (7.5%)	18 (12.3%)	12 (8.2%)
Agree	35 (24.0%)	34 (23.3%)	33 (22.6%)	27 (18.5%)	45 (30.8%)	45 (30.8%)	37 (25.3%)
Strongly agree	8 (5.5%)	9 (6.2%)	29 (19.9%)	5 (3.4%)	25 (17.1%)	4 (2.7%)	1 (0.7%)

**Table 5:** Self-perceived knowledge towards DOACs among study participants.

Knowledge Items	Profession	Disagree *	Unsure	Agree **	Total
I have sufficient knowledge about the perioperative manage of DOACs	Physician	9	14	122	146
	Pharmacist	15	23	119	158
I have sufficient knowledge to inform patients about side effects of DOACs	Physician	11	14	119	146
	Pharmacist	12	14	131	158
I have sufficient to identify and manage drug–drug interactions with DOACs	Physician	8	20	117	146
	Pharmacist	4	20	132	158
I have sufficient knowledge about the contraindications of DOACs	Physician	7	16	121	146
	Pharmacist	7	22	128	158

Note: * Disagree = strongly disagree + disagree. ** Agree = Strongly agree + Agree.

**Table 6:** Self-reported confidence to prescribe (physicians, N = 137) and dispense (pharmacists, N = 132) appropriate doses of DOACs.

Professional Group	Disagree/Strongly Disagree	Unsure	Agree/Strongly Agree	Total
Physicians				
Specialists (cardiologists and IM residents)	8 (9.1%)	8 (9.1%)	72 (81.8%)	88
General practitioners	0 (0.0%)	2 (4.1%)	47 (95.9%)	49
Pharmacists				
Hospital pharmacists	5 (5.2%)	11 (11.5%)	80 (83.3%)	96
Community pharmacists	1 (2.8%)	6 (16.7%)	29 (80.5%)	36

**Table 7:** Participants’ perception towards DOACs vs. vitamin K antagonists.

Questions	Strongly Disagree/Disagree	Unsure	Strongly Agree/Agree	Total N	χ^2^ (df)	*p*-Value
	(Phys */Pharm **)	(Phys/Pharm)	(Phys/Pharm)	(Phys/Pharm)		
DOACs are more effective than vitamin K antagonists	6/5	31/53	99/129	146/158	8.56 (2)	0.014
DOACs cause less intracranial bleeding compared to vitamin K antagonists	5/5	29/65	127/129	146/158	12.04 (2)	0.002
DOACs cause more gastrointestinal bleeding compared to vitamin K antagonists	23/8	20/68	103/106	146/158	22.86 (2)	<0.001
DOACs have fewer drug-drug interactions compared to vitamin K antagonists	12/13	16/29	118/116	146/158	4.28 (2)	0.118
DOACs have fewer side effects compared to vitamin K antagonists	7/13	6/47	134/107	146/158	6.51 (2)	0.038
I feel more confident managing drug-drug interactions with vitamin K antagonists than with DOACs	6/10	65/48	75/100	146/158	9.72 (2)	0.008
I have a personal preference for DOACs over vitamin K antagonists	10/13	10/26	125/119	146/158	6.72 (2)	0.035
Patient using DOACs have higher medication adherence compared to patients using vitamin K antagonists	30/8	21/21	95/129	146/158	14.92 (2)	<0.001
The fact that DOACs do not require INR-monitoring is an advantage	11/7	63/47	60/116	146/158	28.15 (2)	<0.001
Vitamin K antagonists should be preferred in frail elderly patients instead of DOACs	24/8	59/53	59/94	146/158	13.77 (2)	0.001
Vitamin K antagonists should be preferred in patients with high bleeding risk instead of DOACs	17/19	67/57	62/82	146/158	4.33 (2)	0.115
Vitamin K antagonists should be preferred in renal failure patients instead of DOACs	25/15	61/61	54/79	146/158	10.21 (2)	0.006

Note. * Phys = Physician, ** Pharm = Pharmacist.

## Data Availability

The original contributions presented in this study are included in the article. Further inquiries can be directed to the corresponding author.

## Abbreviations

AF	Atrial fibrillation
AI	Artificial intelligence
CDSS	Clinical decision support system
CP	Community pharmacists
DDIs	Drug–drug interactions
DOACs	Direct oral anticoagulants
EHRA	European Heart Rhythm Association Practical Guide
GPs	General practitioners
HCPs	Healthcare professionals
HP	Hospital pharmacists
IP	Internet protocol
IRB	Institutional review board
NVAF	Non-valvular atrial fibrillation
OACs	Oral anticoagulants
SPSS	Statistical Package for the Social Sciences
VKAs	Vitamin K antagonists
VTE	Venous thromboembolism
